# Draft genome sequences of ‘*Candidatus* Chloroploca asiatica’ and ‘*Candidatus* Viridilinea mediisalina’, candidate representatives of the *Chloroflexales* order: phylogenetic and taxonomic implications

**DOI:** 10.1186/s40793-018-0329-8

**Published:** 2018-10-11

**Authors:** Denis S. Grouzdev, Maria S. Rysina, Irina A. Bryantseva, Vladimir M. Gorlenko, Vasil A. Gaisin

**Affiliations:** 1 Institute of Bioengineering, Research Center of Biotechnology of the Russian Academy of Sciences, Moscow, Russian Federation; 2 Winogradsky Institute of Microbiology, Research Center of Biotechnology of the Russian Academy of Sciences, Moscow, Russian Federation; 3 Moscow Institute of Physics and Technology, Moscow, Russian Federation

**Keywords:** Chloroflexi, Chloroflexales, Chloroploca asiatica, Viridilinea mediisalina, Anoxygenic phototrophic bacteria

## Abstract

‘*Candidatus* Chloroploca asiatica’ B7–9 and ‘*Candidatus* Viridilinea mediisalina’ Kir15-3F are mesophilic filamentous anoxygenic phototrophic bacteria from alkaline aquatic environments. Both bacteria became available in the last few years and only in stable enrichment culture. In this study, we report the draft genomic sequences of ‘*Ca*. Chloroploca asiatica’ B7–9 and ‘*Ca.* Viridilinea mediisalina’ Kir15-3F, which were assembled from metagenomes of their cultures with a fold coverage 86.3× and 163.8×, respectively. The B7–9 (5.8 Mb) and the Kir15-3F (5.6 Mb) draft genome harbors 4818 and 4595 predicted protein-coding genes, respectively. In this article, we analyzed the phylogeny of representatives of the *Chloroflexineae* suborder in view of the appearance of new genomic data. These data were used for the revision of earlier published group-specific conserved signature indels and for searching for novel signatures for taxons in the *Chloroflexineae* suborder.

**Electronic supplementary material:**

The online version of this article (10.1186/s40793-018-0329-8) contains supplementary material, which is available to authorized users.

## Introduction

It is difficult to study the mesophilic representatives of filamentous anoxygenic phototrophic (FAP) bacteria (bacteriochlorophyll-based phototrophic Chloroflexota), as maintaining mesophiles in axenic culture and isolating them are challenging. In fact, over the course of four decades of study on FAP bacteria, stable axenic culture of only *Oscillochloris trichoides* DG-6 has been described [1]. Therefore, a description of the mesophiles in enrichment cultures are common in studies [2–6]. However, the approach based on studying the enrichment cultures limits research in frame of morphological observations and rough ecophysiological characterization. Nonetheless, enrichment culture allows for genome sequencing of a target bacterium with high efficiency. Recently, a new mesophilic FAP representative was described in stable highly enriched cultures [5]. Here, we report the results of a genomic study of ‘*Candidatus* Chloroploca asiatica’ B7–9 and a new bacterium, ‘*Candidatus* Viridilinea mediisalina’ Kir15-3F. A partial description of the latter one has been published for the first time. We have assembled high-quality draft genomes of both FAP bacteria. The extended examination into the genomic data was focused on the phylogeny of the *Chloroflexineae* suborder and its taxonomic implications. The new genomic data will help to extend our knowledge about the phylogenetic and functional diversity of FAB bacteria, which is highly limited to date.

## Organism information

### Classification and features

**Fig. 1:**
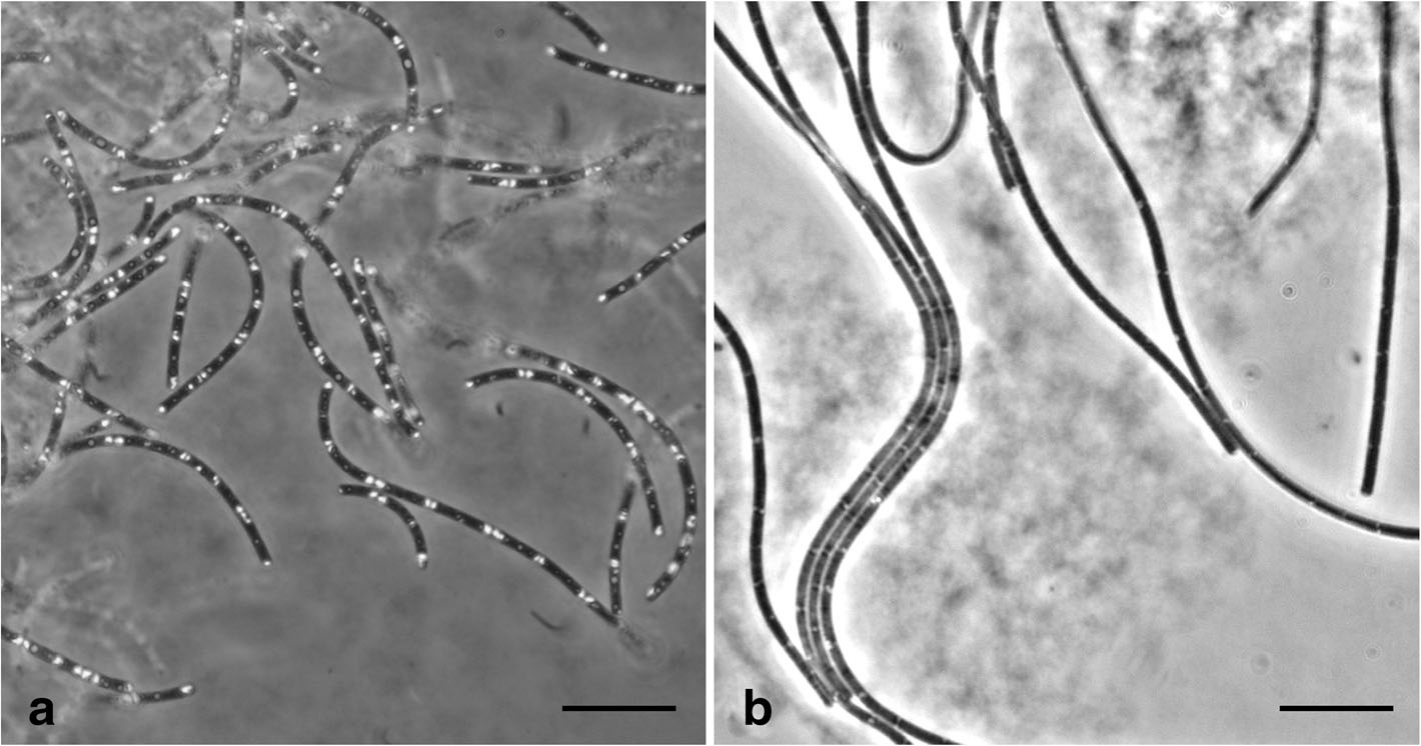
Morphological features of ‘*Candidatus* Chloroploca asiatica’ B7–9 (**a**) and ‘*Candidatus* Viridilinea mediisalina’ Kir15-3F (**b**) as observed on phase-contrast micrographs (Scale bars = 10 μm)

A description of the bacterium ‘*Ca*. Chloroploca asiatica’ was published in 2014 [5]. A partial description of bacterium the ‘*Ca*. Viridilinea mediisalina’ was published in this article. Both bacteria are FAP Chloroflexota bacteria isolated from alkaline environments in Eastern Siberia. The B7–9 was isolated from the Doroninskoe soda lake [5], and the Kir15-3F was isolated from the Kiran soda lake. The bacteria were described in stable enrichment cultures. A summary of the key features of ‘*Ca*. Chloroploca asiatica’ and ‘*Ca*. Viridilinea mediisalina’ is provided in Tables 1 and 2, respectively. Both bacteria have a multicellular filamentous morphology. However, ‘*Ca*. Chloroploca asiatica’ forms short filaments (Fig. 1a) whereas ‘*Ca*. Viridilinea mediisalina’ forms long typical *Oscillochloris*-like filaments (Fig. 1b). The common morphological properties of both bacteria are: a monoderm-type cell envelope, gas vesicles, chlorosomes, polyphosphate-like inclusions and motility (presumably gliding). Both bacteria are supposedly obligate anaerobic anoxygenic phototrophs because they do not grow in the upper part of the agar column and in the dark. Moreover, both bacteria are mesophiles and exhibit the best growth under alkaline conditions.

**Table 1:** Classification and general characteristics of ‘*Ca.* Chloroploca asiatica*’* B7–9 [25]

MIGS ID	Property	Term	Evidence code^a^
	Classification	Domain: *Bacteria*	TAS [26]
		Phylum: *Chloroflexota*	TAS [27–29]
		Class: *Chloroflexia*	TAS [14, 30]
		Order: *Chloroflexales*	TAS [14, 30]
		Family: *incertae sedis*	IDA
		Genus: ‘*Ca*. Chloroploca’	TAS [5]
		Species: ‘*Ca*. Chloroploca asiatica’	TAS [5]
		Strain B7–9	TAS [5]
	Gram stain	Negative	TAS [5]
	Cell shape	Filaments	TAS [5]
	Motility	Motile	TAS [5]
	Sporulation	Not reported	NAS
	Temperature range	Not determined	TAS [5]
	Optimum temperature	25–32 °C	TAS [5]
	pH range; Optimum	Not determined; 8.0	TAS [5]
	Carbon source	Not determined	TAS [5]
MIGS-6	Habitat	Soda lakes	TAS [5]
MIGS-6.3	Salinity	0.3–1.5% NaCl (*w*/*v*)	TAS [5]
MIGS-22	Oxygen requirement	Anaerobic	TAS [5]
MIGS-15	Biotic relationship	Free-living	TAS [5]
MIGS-14	Pathogenicity	Non-pathogen	NAS
MIGS-4	Geographic location	Russia/East Siberia	TAS [5]
MIGS-5	Sample collection	September 2010–2012	TAS [5]
MIGS-4.1	Latitude	51.235707	TAS [5]
MIGS-4.2	Longitude	112.236169	TAS [5]
MIGS-4.4	Altitude	Not determined	TAS [5]

^a^Evidence codes - IDA: Inferred from Direct Assay; TAS: Traceable Author Statement (i.e., a direct report exists in the literature); NAS: Non-traceable Author Statement (i.e., not directly observed for the living, isolated sample, but based on a generally accepted property for the species, or anecdotal evidence). These evidence codes are from the Gene Ontology project [31, 32]

**Table 2:** Classification and general characteristics of ‘*Ca.* Viridilinea mediisalina*’* Kir15-3F [25]

MIGS ID	Property	Term	Evidence code^a^
	Classification	Domain: *Bacteria*	TAS [26]
		Phylum: *Chloroflexota*	TAS [27–29]
		Class: *Chloroflexia*	TAS [14, 30]
		Order: *Chloroflexales*	TAS [14, 30]
		Family: *incertae sedis*	IDA
		Genus: ‘*Ca*. Viridilinea’	IDA
		Species: ‘*Ca*. Viridilinea mediisalina’	IDA
		Strain Kir15-3F	IDA
	Gram stain	Not determined	IDA
	Cell shape	Filaments	IDA
	Motility	Motile	IDA
	Sporulation	Not reported	IDA
	Temperature range	Mesophile	IDA
	Optimum temperature	Not determined	IDA
	pH range; Optimum	Not determined	IDA
	Carbon source	Not determined	IDA
MIGS-6	Habitat	Soda lakes	IDA
MIGS-6.3	Salinity	Halotolerant	IDA
MIGS-22	Oxygen requirement	Anaerobic	IDA
MIGS-15	Biotic relationship	Free-living	IDA
MIGS-14	Pathogenicity	Non-pathogen	NAS
MIGS-4	Geographic location	Russia/East Siberia	IDA
MIGS-5	Sample collection	September 2015	IDA
MIGS-4.1	Latitude	50.332958	IDA
MIGS-4.2	Longitude	106.851128	IDA
MIGS-4.4	Altitude	Not determined	IDA

^a^Evidence codes - IDA: Inferred from Direct Assay; TAS: Traceable Author Statement (i.e., a direct report exists in the literature); NAS: Non-traceable Author Statement (i.e., not directly observed for the living, isolated sample, but based on a generally accepted property for the species, or anecdotal evidence). These evidence codes are from the Gene Ontology project [31, 32]

**Fig. 2:**
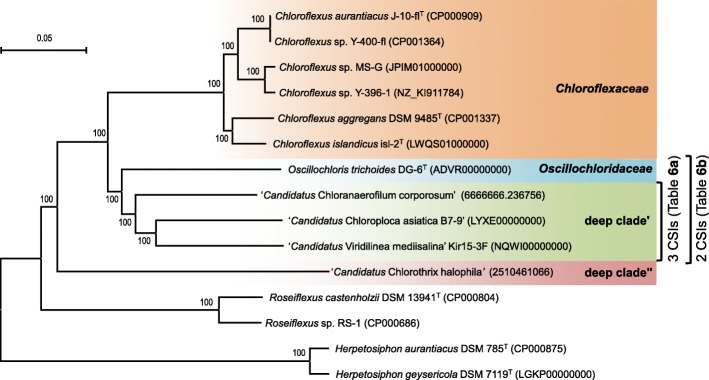
Neighbor-joining phylogenetic tree inferred from the concatenated amino acid sequences of the core proteins showing the phylogeny of the representatives of the *Chloroflexineae* suborder. A total of 52,793 positions in the final dataset were analyzed. The bootstrap values obtained with the neighbor-joining methods are based on 1000 replicates. The scale bars represent a 5% amino acid sequence divergence. The accession number for ‘*Ca*. Chloranaerofilum corporosum’ corresponds to RAST, and the accession number for ‘*Ca*. Chlorothrix halophila*’* corresponds to IMG database. The other accession numbers correspond to GenBank

Phylogenetic analysis based on the concatenated amino acid sequences of the core proteins revealed that ‘*Ca*. Chloroploca asiatica’ B7–9 and ‘*Ca*. Viridilinea mediisalina’ Kir15-3F are closest relatives to each other (Fig. 2). The closest taxonomically defined representative for the clade of both bacteria is the mesophilic bacterium *O. trichoides* DG-6. However, the closest relative is ‘*Ca.* Chloranaerofilum corporosum’, whose population has been detected in the Mushroom hot spring [7]. All four bacteria were assigned to the *Chloroflexales* order, which encompasses all representatives of the FAP bacteria. However, the complete taxonomic position of ‘*Ca*. Chloroploca asiatica’ and ‘*Ca*. Viridilinea mediisalina’ as well as ‘*Ca.* Chloranaerofilum corporosum’ remains unclear.

#### Chemotaxonomic data

Bacteriochlorophyll *c* is the main phototrophic pigment of both ‘*Ca*. Chloroploca asiatica’ and ‘*Ca*. Viridilinea mediisalina’, and bacteriochlorophyll *a* is found in trace amounts [5].

## Genome sequencing information

### Genome project history

The study of ‘*Candidatus* Chloroploca asiatica’ B7–9 and ‘*Candidatus* Viridilinea mediisalina’ Kir15-3F was conducted as part of the collaborative project of the Laboratory of Molecular Diagnostics and Laboratory of Ecology and Geochemical Activity of Microorganisms at the Research Center for Biotechnology RAS (Moscow, Russian Federation). Attempts to isolate the axenic culture of both bacteria have not been successful. Therefore, to further study these bacteria, a strategy based on metagenomic sequencing of their highly enriched culture was used. We assembled a high-quality draft genome sequence of the target bacteria with a fold coverage of more than 86.3×. The draft genome sequences have been deposited in GenBank under the accession numbers LYXE00000000.1 and NQWI00000000.1 for B7–9 and Kir15-3F, respectively. The main project information is summarized in Table 3.

**Table 3:** Project information

MIGS ID	Property	‘*Ca*. Chloroploca asiatica’ B7–9	‘*Ca*. Viridilinea mediisalina’ Kir15-3F
		Term	Term
MIGS 31	Finishing quality	Improved high-quality draft	Improved high-quality draft
MIGS-28	Libraries used	Illumina Standard shotgun library	Illumina Standard shotgun library
MIGS 29	Sequencing platforms	Illumina Hiseq 2500	Illumina Hiseq 2500
MIGS 31.2	Fold coverage	86.3×	163.8×
MIGS 30	Assemblers	SPAdes v. 3.11.1	SPAdes v. 3.11.1
MIGS 32	Gene calling method	RAST, PGAP	RAST, PGAP
	Locus Tag	A9Q02	CJ255
	Genbank ID	LYXE00000000.1	NQWI00000000.1
	GenBank Date of Release	12-OCT-2017	12-OCT-2017
	GOLD ID	Gp0236327	Gp0236326
	BIOPROJECT	PRJNA323704	PRJNA398606
MIGS 13	Source Material Identifier	B7–9	Kir15-3F
	Project relevance	Evolution of FAP bacteria	Evolution of FAP bacteria

### Growth conditions and genomic DNA preparation

‘*Ca*. Chloroploca asiatica’ B7–9 was grown in an agar medium described previously [5] in glass tubes at 27 °C in the light (3100 lx). The bacterium forms spherical colonies, which were used for isolation of the total genomic DNA. In the first step, the colonies were collected from the agar into a 2.0-ml screw-cap microcentrifuge tube containing 375 μl of TE buffer (containing 10 mM Tris and 1 mM EDTA) and 1-mm glass beads to make a total volume of about 500 μl. The microcentrifuge tube was treated using a Mini-Beadbeater (Biospec) until two to three cells were observed to have formed filaments under the microscope. The 475 μl suspension was transferred to a 1.5-ml microcentrifuge tube, to which 25 μl of 100 mM Dithiothreitol was added, mixed and incubated for 100 min at 65 °C. Following this, add 100 μl of 10% Sodium dodecyl sulfate and 5 μl Proteinase K (20 mg/mL). were added, mixed in, and incubated for 60 min at 37 °C. Next, 100 μl of 5 M NaCl and 5 μl RNAse (10 mg/mL) were added, mixed and incubated for 10 min at 65 °C. Finally, 160 μl of Cetrimonium bromide (CTAB) solution (containing 5% CTAB and 0.35 M NaCI) was added, mixed, and incubated for 10 min at 65 °C. The solution was allowed to cool down to room temperature, after which 700 μl of chloroform was mixed in carefully and the solution was spun for 10 min in a microcentrifuge. The upper phase was transferred to a fresh microcentrifuge tube and the interface was left behind. These procedures were repeated with chloroform, and then the upper phase was transferred to a fresh tube. Isopropanol (0.6 vol.) was added to precipitate the DNA. The precipitated DNA was washed with 70% ethanol, briefly dried, and redissolved in MQ water.

‘*Ca*. Viridilinea mediisalina’ Kir15-3F was grown on agar that covered the bottom of a 50-ml vial filled with liquid medium consisting of the following mix (per litre): KH_2_PO_4_ (0.20 g), NH_4_Cl (0.20 g), MgCl_2_·6H_2_O (0.20 g), KCl (0.30 g), NaCl (25.0 g), Na_2_S_2_O_3_ (0.30 g), Na_2_SO_4_ (0.30 g), CaCl_2_·2H_2_O (0.05 g), NaHCO_3_ (0.60 g), Na_2_S·9H_2_O (0.70 g), soytone (0.05 g), yeast extract (0.05 g), sodium acetate (0.10 g), trace element solution (1 mL) and Pfennig’s vitamin solution (1 mL). The final pH was adjusted to 9.0. The vial was incubated at 38 °C in the light (3800 lx). ‘*Ca*. Viridilinea mediisalina’ Kir15-3F was isolated from the biofilm at the bottom. The biofilm was collected for isolation of the genomic DNA, following the same protocol as described above for ‘*Ca*. Chloroploca asiatica’ B7–9.

### Genome sequencing and assembly

The same method was used for sequencing of the total DNA from the cultures of both bacteria. The sequencing was performed at “I gene” LLC, Moscow, Russian Federation. A sequence library was constructed with the NEBNext DNA library prep reagent set for Illumina according to the manufacturer’s protocol. The 4,000,203 and 4,793,690 paired-end 150-bp reads were generated using Illumina Hiseq 2500 platforms for metagenomic sequences of the B7–9 and Kir15-3F culture, respectively. Raw sequences were assembled with SPAdes version 3.11.1 [8] and binned using MetaWatt version 3.5.3 [9]. The Chloroflexota genomes were uploaded to RAST [10] for overall characterization and were assessed for completeness and contamination using CheckM [11]. Finally, they were assembled into 166 and 291 contigs for ‘*Ca*. Chloroploca asiatica’ B7–9 (coverage, 86.3×) and ‘*Ca*. Viridilinea mediisalina’ Kir15-3F (coverage, 163.8×) bacterium, respectively.

### Genome annotation

The assembled draft genomic sequences of ‘*Ca*. Chloroploca asiatica’ B7–9 and ‘*Ca*. Viridilinea mediisalina’ Kir15-3F were submitted to the NCBI Prokaryotic Genome Annotation Pipeline for annotation [12].

## Genome properties

The properties of both genomes are summarized in Table 4. The draft genome of ‘*Ca*. Chloroploca asiatica’ B7–9 contained 5,817,919 bp with a G + C content of 58.81% and 4878 (100%) predicted genes: 4818 (98.77%) were protein-coding genes; 46 (0.94%), tRNA genes; and 9 (0.18%), rRNA genes. The genome was 98.74% complete. The draft genome of ‘*Ca*. Viridilinea mediisalina’ Kir15-3F contained 5,588,620 bp with a G + C content of 58.01% and 4657 (100%) predicted genes: 4595 (98.67%) were protein-coding genes; 46 (0.99%), tRNA genes; and 10 (0.21%), rRNA genes. This genome was 99.21% complete. The properties and statistics of both genomes are summarized in Table 4. The assignment of genes to COG functional categories is presented in Table 5.

**Table 4:** Genome statistics

Attribute	‘*Ca*. Chloroploca asiatica’ B7–9		‘*Ca*. Viridilinea mediisalina’ Kir15-3F	
	Value	% of Total	Value	% of Total
Genome size (bp)	5,817,919	100.00	5,588,620	100.00
DNA coding (bp)	5,229,318	89.88	4,851,981	86.82
DNA G + C (bp)	3,421,494	58.81	3,241,714	58.01
DNA scaffolds	166	100.00	291	100.00
Total genes	4878	100.00	4657	100.00
Protein coding genes	4818	98.77	4595	98.67
RNA genes	60	1.23	62	1.33
Pseudo genes	–	–	–	–
Genes in internal clusters	1117	22.90	1081	23.21
Genes with function prediction	3518	72.12	3234	69.44
Genes assigned to COGs	2634	54.00	2356	50.59
Genes with Pfam domains	3550	72.78	3264	70.09
Genes with signal peptides	171	3.51	139	2.98
Genes with transmembrane helices	1371	28.11	1127	24.20
CRISPR repeats	95		445

**Table 5:** Number of genes associated with general COG functional categories

Code	‘*Ca*. Chloroploca asiatica’ B7–9		‘*Ca*. Viridilinea mediisalina’ Kir15-3F		Description
	Value	%age	Value	%age	
J	178	6.05	178	6.79	Translation, ribosomal structure and biogenesis
A	–	–	–	–	RNA processing and modification
K	163	5.54	144	5.49	Transcription
L	105	3.57	110	4.19	Replication, recombination and repair
B	2	0.07	1	0.04	Chromatin structure and dynamics
D	43	1.46	36	1.37	Cell cycle control, Cell division, chromosome partitioning
V	74	2.52	81	3.09	Defense mechanisms
T	215	7.31	248	9.45	Signal transduction mechanisms
M	225	7.65	207	7.89	Cell wall/membrane biogenesis
N	24	0.82	24	0.91	Cell motility
U	29	0.99	32	1.22	Intracellular trafficking and secretion
O	140	4.76	136	5.18	Posttranslational modification, protein turnover, chaperones
C	193	6.56	154	5.87	Energy production and conversion
G	198	6.73	135	5.03	Carbohydrate transport and metabolism
E	254	8.64	202	7.70	Amino acid transport and metabolism
F	83	2.82	69	2.63	Nucleotide transport and metabolism
H	191	6.50	163	6.21	Coenzyme transport and metabolism
I	128	4.35	104	3.96	Lipid transport and metabolism
P	150	5.10	142	5.41	Inorganic ion transport and metabolism
Q	61	2.07	39	1.49	Secondary metabolites biosynthesis, transport and catabolism
R	307	10.44	280	10.67	General function prediction only
S	127	4.32	110	4.19	Function unknown
–	2244	46.00	2301	49.41	Not in COGs

The total is based on the total number of protein coding genes in the genome

## Insights from the genome sequence

The draft genomes reported here and the recently published partial genomic sequence of ‘*Ca.* Chloranaerofilum corporosum’ [13] provide a detailed picture of the evolutionary relationships among chlorosome-containing representatives. Recently, it was proposed that the *Chloroflexales* order of the class *Chloroflexia* be divided into two suborders: *Roseiflexineae* and *Chloroflexineae*. It was proposed that the first one encompasses chlorosome-lacking *Roseiflexus* spp., whereas the other one encompasses all the chlorosome-containing representatives of the order [14]. This suggestion based on the obvious morphophysiological differences is strongly supported by the results of genomic analysis [15]. However, the taxonomic hierarchy within *Chloroflexineae* is not so clear, and this is why it was a subject of the current analysis.

### Group-specific conserved signature indels

In the first step, we analyzed the previously proposed specific conserved signature indels (CSIs) [14]. Analysis revealed that ‘*Ca*. Chloroploca asiatica’ B7–9, ‘*Ca*. Viridilinea mediisalina’ Kir15-3F and ‘*Candidatus* Chloranaerofilum corporosum’ have *Chloroflexinea*-specific insertions in a Phage SPO1 DNA pol-like protein, nucleoside diphosphate kinase, translation initiation factor-2, threonine synthase, ArsA and the acetolactate synthase large subunit, which have been reported previously [14]. Thus, this finding confirms that the bacteria belong to the *Chloroflexinea* suborder. However, the new chlorosome-containing FAP bacteria do not have specific inserts in the protein sequences of a nucleotide sugar dehydrogenase (Additional file 1: Figure S1a). Moreover, the new representatives have specific insertion in the magnesium-protoporphyrin IX monomethyl ester cyclase proteins (Additional file 1: Figure S1b) was earlier proposed to be *Chloroflexus*-specific CSIs [14]. Thus, the new genomic data indicate that the CSIs based on the nucleotide sugar dehydrogenase and magnesium-protoporphyrin IX monomethyl ester cyclase proteins must be eliminated from the taxonomic description.

In the second step, we identified new specific CSIs for the studied bacteria ‘*Ca*. Chloroploca asiatica’ B7–9, ‘*Ca*. Viridilinea mediisalina’ Kir15-3F and ‘*Candidatus* Chloranaerofilum corporosum’: specifically, CSIs for phosphoglycerate kinase, heat-inducible transcription repressor, and UMP kinase were identified (Additional file 1: Figure S2a-c). Moreover, some of the new CSIs were found to be common to both the new bacteria and *O. trichoides* DG-6: these were threonine synthase and glutamate 5-kinase (Additional file 1: Figure S3a and b). The new CSIs are shown in Table 6.

**Table 6:** CSIs that are specific for *O. trichoides*, ‘*Ca*. Chloranaerofilum corporosum’, *O. trichoides*, ‘*Ca*. Chloroploca asiatica’ and ‘*Ca*. Viridilinea mediisalina’

Protein name	GI number	Indel size	Indel position
(a) CSIs that are specific for the Viridilinea + Chloroploca + Chloranaerofilum			
Phosphoglycerate kinase	WP_044200294.1	1 aa Ins	54–55
Heat-inducible transcription repressor HrcA	WP_006560707.1	1 aa Ins	131–132
UMP kinase	WP_006562130.1	1 aa Del	23
(b) CSIs that are specific for the Viridilinea + *Oscillochloris* + Chloroploca + Chloranaerofilum			
Threonine synthase	WP_006561465.1	1 aa Del	304–305
Glutamate 5-kinase	WP_044201831.1	2 aa Ins	65–66

### Phylogeny of the *Chloroflexineae* suborder

The concatenated core protein tree has strong bootstrap support for the observed branching (Fig. 2), with the chlorosome-containing FAP bacteria represented as a monophyletic group relative to the chlorosome-lacking *Roseiflexus* spp. This core protein phylogeny, similarity in pigment composition and the presence of common CSIs indicate that the *Chloroflexineae* suborder encompasses the *Chloroflexus* strains, ‘*Ca.* Chloranaerofilum corporosum’, ‘*Ca*. Chlorothrix halophila’, *O. trichoides*, ‘*Ca*. Chloroploca asiatica’ and ‘*Ca.* Viridilinea mediisalina’ i.e., all the chlorosome-containing FAP bacteria. Bacteria from the *Roseiflexineae* suborder show ancestral relations to the representatives listed above [15]. The tree depicts three main clades within the *Chloroflexineae* suborder: the clade of closely related *Chloroflexus* species, the ‘*Ca*. Chlorothrix halophila’ clade and the clade containing the deeply branched lineages ‘*Ca*. Chloranaerofilum corporosum’, *O. trichoides*, ‘*Ca*. Chloroploca asiatica’ and ‘*Ca*. Viridilinea mediisalina’.

Strains of the *Chloroflexus* genus form a clade that is clearly separated from the other representatives of the suborder (Fig. 2). This branching has congruence with the morphological and ecophysiological uniformity of *Chloroflexus* strains, which are thermophilic photoheterotrophs capable of respiration in the dark [16–18]. Genomes of the *Chloroflexus* strains contain genes of the autotrophic 3-hydroxypropionate CO_2_ fixation cycle (3-OHP cycle), the activity of which was demonstrated in the OK-70-fl strain [19]. At the moment, only thermophilic *Chloroflexus* species form the *Chloroflexaceae* family. A mesophilic *Chloroflexus*-like bacterium, called ‘*Cfl*. *aurantiacus* var. *mesophilus*’, was identified based on its morphological properties [20]. However, since 16S rRNA gene sequence and other sequencing data are absent, it is highly likely that this bacterium does not belong to the 16S rRNA *Chloroflexus* clade.

The next two clades were formed by genera represented by a single species. The first clade, which is comprised of the halophilic bacterium ‘*Ca*. Chlorothrix halophila’, forms a deeply branched lineage within the chlorosome-containing group in accordance with the protein phylogenetic tree (Fig. 2). It was speculated earlier that significant deep branching of a protein tree can be the result of adaptation to halophilic conditions [15]. This led to the preferential use of the 16S phylogeny, but this created contradictions in the protein tree. This explains the difficulty with using the CSI approach for this bacterium. However, ‘*Ca*. Chlorothrix halophila’ clearly formed an external deeply branched lineage in the current core protein tree (Fig. 2). Moreover, this bacterium has a 14–18% dissimilarity, which represents is the greatest distance from other representatives of the *Chloroflexineae* suborder according to a comparison of the 16S rRNA sequences. The bacterium ‘*Ca*. Chlorothrix halophila’ shows preference for halophilic conditions, which is a unique characteristic among the described FAP bacteria [3]. The halophilic preference, combined with the results of phylogenetic analysis and cell ultrastructure, indicate that the bacterium is a candidate for a rank not below the family level.

The third clade was formed by *O. trichoides* and the recently described bacteria ‘*Ca*. Chloranaerofilum corporosum’, ‘*Ca*. Chloroploca asiatica’ and ‘*Ca*. Viridilinea mediisalina’. The bacterium *O. trichoides* DG-6 is a type genus and species for *Oscillochloridaceae* family [21]. Main specific features of the *O. trichoides* strains are mesophilic lifestyle, the presence of gas vesicles, autotrophic Calvin cycle CO_2_ fixation (the 3-OHP cycle is absent), and of nitrogen fixation. It was proposed that *Chloronema* species belonged to the *Oscillochloridaceae* family [14], but physiological and sequence data for this bacterium remain highly limited. The bacteria ‘*Ca*. Chloroploca asiatica’ and ‘*Ca*. Viridilinea mediisalina’ have some common features with *O. trichoides*, such as their mesophilic features, the presence of gas vesicles, motility, and inability for growth in aerobic conditions and in the dark [1, 5]. However, the closest relative to both new bacteria is the probably thermophilic bacterium ‘*Ca*. Chloranaerofilum corporosum’ (Fig. 2). The delineation of the subclades ‘Chloroploca+Viridilinea’ and ‘*Ca*. Chloranaerofilum corporosum’ from *O. trichoides* is supported by the CSIs identified (Additional file 1: Figure S2a-c). Additionally, the three recently described bacteria have 3-OHP cycle genes and lack Calvin cycle genes.

The deep divergence between the subclades *O. trichoides* and ‘Chloroploca+Viridilinea’ was supported by the results of an analysis of the average amino acid identity and percentage of conserved proteins. AAI was calculated using a web-based tool [22], and POCP was calculated using a script described previously with some modifications [23]. The modified script was published at figshare.com [24]. The results for ‘*Ca*. Chloranaerofilum corporosum’ should be considered carefully because of the low completeness of the genome (64%) [7], which could lead to misinterpretation, particularly with regard to POCP. The AAI values for ‘*Ca*. Chloranaerofilum corporosum’ could be overestimated due to the presence of ambiguous amino acids. The 2999 “X” residues were found in a set of all proteins from the genome. Therefore, we will further focus on AAI and PCOP in the comparison of the subclades *O. trichoides* and ‘Chloroploca+Viridilinea’.

**Fig. 3:**
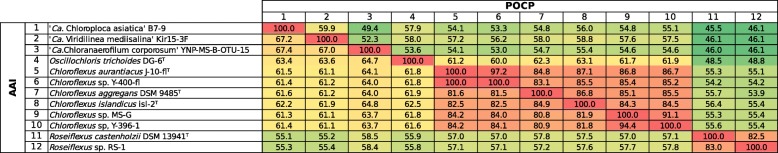
Results of the analysis of average amino acid identity (AAI) and percentage of conserved proteins (POCP)

On the one hand, the highest AAI value, about 67, was found for ‘*Ca*. Chloroploca asiatica’, ‘*Ca*. Viridilinea mediisalina’ and ‘*Ca*. Chloranaerofilum corporosum’ (Fig. 3). On the other hand, the values between the subclades *O. trichoides* and ‘Chloroploca+Viridilinea’ were about 63, which is close to the interfamily values for the *Chloroflexaceae* and *Oscillochloridaceae* families (61.6–62.5). Moreover, the POCP values were close to those between the subclades *O. trichoides* and ‘Chloroploca+Viridilinea’ (57.9–58.0) and between the clades of the *Chloroflexaceae* and *Oscillochloridaceae* families (60.0–63.1). These results provide evidence that the listed subclades have significant phylogenetic divergence which corresponds to family-level difference within *Chloroflexineae* suborder.

The low genomic completeness of the bacterium ‘*Ca*. Chloranaerofilum corporosum’ limited the pan-genomic comparison and search for CSIs. Nonetheless, it is clear that this bacterium is the closest relative to the subclade ‘Chloroploca+Viridilinea’, based on the results of the phylogenetic analysis and the common CSIs identified (Fig. 2, Additional file 1: Figure S2a-c). The phylogenetic distance is significant according to both the core protein tree and 16S rRNA phylogeny. Importantly, ‘*Ca*. Chloranaerofilum corporosum’ has distinctive ecophysiological and morphological features. The bacterium forms a native population within the 52.5 °C temperature zone of the Mushroom hot spring [13]. Additionally, gas vesicles were not shown. However, the features were described using environmental observations, and therefore, experimental verification is required. At the moment it is difficult to make an exact taxonomic proposal: Does ‘Chloroploca+Viridilinea’ represent a new family within the *Chloroflexineae* suborder or not?

## Conclusions

Comparative analysis of the new genome of recently described chlorosome-containing FAP bacteria exhibits a trend towards the segregation of new families within the *Chloroflexineae* suborder. If representatives of the *Chloroflexaceae* family show phylogenetic uniformity, other bacteria from the *Chloroflexineae* suborder significantly diverge from each other. The observed “phylogenetic jumps” among lineages within the *Chloroflexineae* suborder could reflect high underestimation of the genomic diversity of FAP bacteria.

## Additional file

Additional file 1: **Figure S1.** Previously reported CSIs: nucleotide sugar dehydrogenase (a) and magnesium-protoporphyrin IX monomethyl ester cyclase (AcsF) proteins (b). **Figure S2.** CSIs which specific for ‘*Ca*. Chloroploca asiatica’ B7–9, ‘*Ca*. Viridilinea mediosalina’ Kir15-3F and ‘*Candidatus* Chloranaerofilum corporosum’: phosphoglycerate kinase (a), heat-inducible transcription repressor (b), UMP kinase (c). **Figure S3.** CSIs which specific for ‘*Ca*. Chloroploca asiatica’ B7–9, ‘*Ca*. Viridilinea mediosalina’ Kir15-3F, ‘*Candidatus* Chloranaerofilum corporosum’ and *O*. *trichoides* DG-6: threonine synthase (a) and glutamate 5-kinase (b). (PDF 1652 kb)

## Abbreviation

FAP: filamentous anoxygenic phototrophic
