# Supplementary material for: Draft genome sequences of ‘Candidatus Chloroploca asiatica’ and ‘Candidatus Viridilinea mediisalina’, candidate representatives of the Chloroflexales order: phylogenetic and taxonomic implications
Source: Stand Genomic Sci. 2018 Oct 11;13:24. doi: 10.1186/s40793-018-0329-8 (PMC6180586; doi:10.1186/s40793-018-0329-8)
Supplement: Supplementary file 1 — Figure S1. Previously reported CSIs: nucleotide sugar dehydrogenase (a) and magnesium-protoporphyrin IX monomethyl ester cyclase (AcsF) proteins (b). Figure S2. CSIs which specific for ‘Ca. Chloroploca asiatica’ B7–9, ‘Ca. Viridilinea mediosalina’ Kir15-3F and ‘Candidatus Chloranaerofilum corporosum’: phosphoglycerate kinase (a), heat-inducible transcription repressor (b), UMP kinase (c). Figure S3. CSIs which specific for ‘Ca. Chloroploca asiatica’ B7–9, ‘Ca. Viridilinea mediosalina’ Kir15-3F, ‘Candidatus Chloranaerofilum corporosum’ and O. trichoides DG-6: threonine synthase (a) and glutamate 5-kinase (b). (PDF 1652 kb) [file 40793_2018_329_MOESM1_ESM.pdf]

Supplementary figures

Draft genome sequences of '*Candidatus* Chloroploca asiatica' and '*Candidatus* Viridilinea mediisalina', as representatives of the *Chloroflexales* order: Phylogenetic and taxonomic implications.

Denis S Grouzdev, Maria S. Rysina, Irina A Bryantseva, Vladimir M Gorlenko and Vasil A. Gaisin*

* Correspondence: vasil.beingi@gmail.com

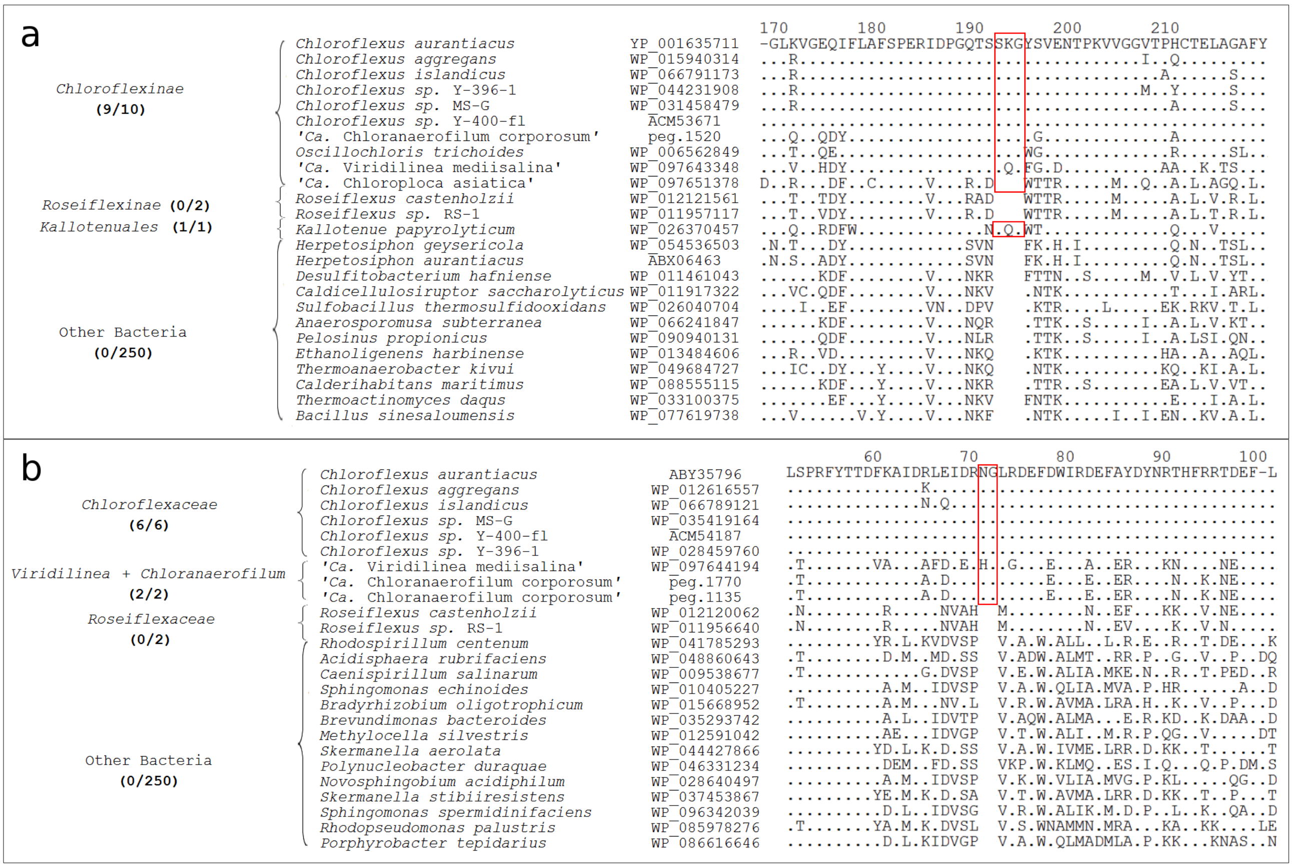

Figure 1S. Previously reported CSIs: nucleotide sugar dehydrogenase (a) and magnesium-protoporphyrin IX monomethyl ester cyclase (AcsF) proteins (b).

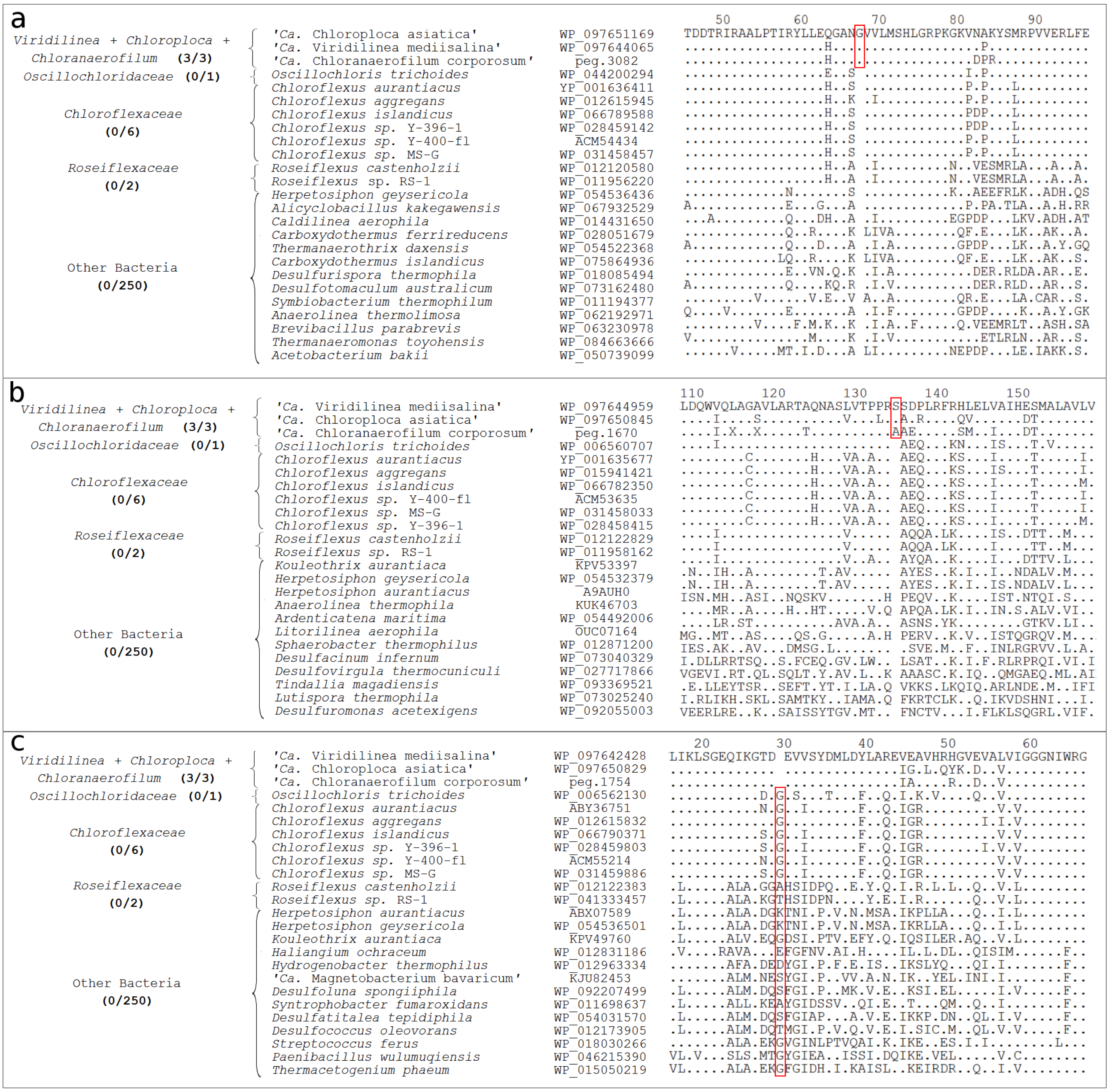

Figure 2S. CSIs which specific for ‘*Ca*. Chloroploca asiatica’ B7-9, ‘*Ca*. Viridilinea mediosalina’ Kir15-3F and ‘*Candidatus* Chloranaerofilum corporosum’: phosphoglycerate kinase (a), heat-inducible transcription repressor (b), UMP kinase (c).

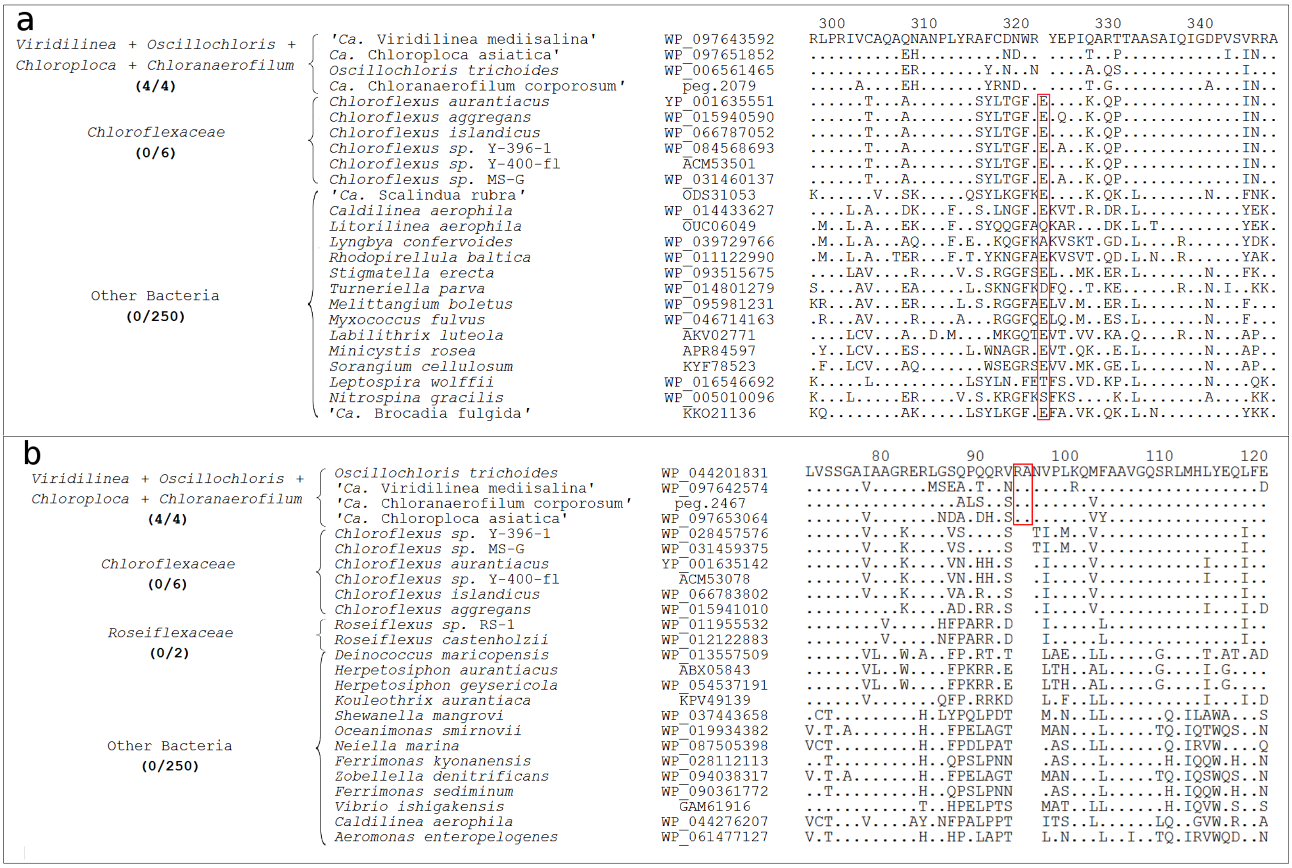

Figure 3S. CSIs which specific for ‘*Ca*. Chloroploca asiatica’ B7-9, ‘*Ca*. Viridilinea mediosalina’ Kir15-3F, ‘*Candidatus* Chloranaerofilum corporosum’ and *O. trichoides* DG-6: threonine synthase (a) and glutamate 5-kinase (b).
